# Design, Synthesis, and Biomedical Application of Multifunctional Fluorescent Polymer Nanomaterials

**DOI:** 10.3390/molecules28093819

**Published:** 2023-04-29

**Authors:** Qingpan Bu, Ping Li, Yunfei Xia, Die Hu, Wenjing Li, Dongfang Shi, Kai Song

**Affiliations:** 1 School of Life Science, Changchun Normal University, Changchun 130032, China; buqingpan@ccsfu.edu.cn (Q.B.); chunlps@163.com (P.L.); xiayunfei1103@163.com (Y.X.); hudie18784775519@163.com (D.H.); 2 School of Education, Changchun Normal University, Changchun 130032, China; liwenj678@163.com; 3 Institute of Science, Technology and Innovation, Changchun Normal University, Changchun 130032, China

**Keywords:** light-emitting polymer nanomaterials, rare earth polymers, semiconducting polymers, organic fluorescent small molecule cell imaging, biomedical imaging

## Abstract

Luminescent polymer nanomaterials not only have the characteristics of various types of luminescent functional materials and a wide range of applications, but also have the characteristics of good biocompatibility and easy functionalization of polymer nanomaterials. They are widely used in biomedical fields such as bioimaging, biosensing, and drug delivery. Designing and constructing new controllable synthesis methods for multifunctional fluorescent polymer nanomaterials with good water solubility and excellent biocompatibility is of great significance. Exploring efficient functionalization methods for luminescent materials is still one of the core issues in the design and development of new fluorescent materials. With this in mind, this review first introduces the structures, properties, and synthetic methods regarding fluorescent polymeric nanomaterials. Then, the functionalization strategies of fluorescent polymer nanomaterials are summarized. In addition, the research progress of multifunctional fluorescent polymer nanomaterials for bioimaging is also discussed. Finally, the synthesis, development, and application fields of fluorescent polymeric nanomaterials, as well as the challenges and opportunities of structure–property correlations, are comprehensively summarized and the corresponding perspectives are well illustrated.

## 1. Introduction

Bioluminescent imaging is a visual imaging method that detects the intrinsic fluorescence of organisms, or the intensity of fluorescence luminescence after fluorescent materials mark organisms. Compared with traditional medical diagnostic imaging methods, it has the characteristics of fast imaging speed, high resolution, and applying no radiation damage to organisms. Applications can range from micron-sized cells to large-sized living organisms [1,2,3]. After years of research and development, commercial bioluminescence imaging systems are now widely used in the biomedical field, including laser scanning confocal microscopes, two-photon laser scanning microscopy imaging systems, and in vivo fluorescence imaging techniques. In combination with traditional biological imaging techniques such as magnetic resonance imaging (MRI), ultrasonic imaging (US), and computed tomography (CT), etc., the functions are complementary, and more accurate and effective biological imaging can be achieved, providing a reliable imaging method for the early diagnosis of cancer [4,5,6].

At present, fluorescent imaging materials mainly include inorganic fluorescent functional materials (quantum dots, rare earth luminescent materials, noble metal nanomaterials, etc.) and organic fluorescent functional materials (organic small molecule fluorescent materials and semiconducting polymers) [7,8,9]. Among them, quantum dots have good fluorescence quantum efficiency and photostability, but cannot avoid the biological toxicity of heavy metals [10,11]. Organic small molecule fluorescent dyes are the most widely used class of fluorescent materials. However, due to the disadvantages of poor stability, easy photobleaching, small Stokes shift and short fluorescence lifetime, its application range is greatly limited [12]. Rare earth luminescent materials, compared with other fluorescent polymer materials, have a narrow emission band (10–20 nm) and a large Stokes shift. The emission lifetime from microseconds to milliseconds can be achieved, which greatly reduces the interference of the self-luminous background of biological tissues. However, the material itself has poor biocompatibility, and it is prone to fluorescence aggregation quenching or quenching by water in a physiological environment [13,14]. Semiconducting polymers also have many excellent characteristics, for example, good photostability and photothermal performance, easy functional modification of the surface of the material, and good biocompatibility. However, the molecular weight is not easy to control during the preparation process, and the metabolic mechanism in the human body is still unclear [15,16].

Fluorescent polymer nanomaterials are prepared by physically doping or chemically bonding amphiphilic block polymers and fluorescent functional materials [17,18]. The composite material has the characteristics of good optical stability and wide application range of functional fluorescent materials. Additionally, it is also possible to select polymer monomers with different functions; while retaining the good water solubility and excellent biocompatibility of polymer nanomaterials, the controllable synthesis of properties such as size, morphology, stability, and surface properties can be achieved [19,20]. The complementary advantages of the two provide new ideas for expanding the application range of fluorescent functional materials in the field of biomedicine and have attracted the extensive attention of scientific researchers [21,22,23].

In summary, this paper discusses the functionalization strategy of fluorescent polymers and the preparation methods for fluorescent polymer nanomaterials. It also systematically introduces the research and development status and application prospects of fluorescent polymer nanomaterials based on rare earth luminescent materials, semiconducting polymers, and small organic molecules from recent years. It is emphasized that the design and application of fluorescent polymer nanomaterials should be functionalized from the perspective of synthesis and optimization according to need. Finally, the development direction and challenges of polymer nanomaterials in the fields of optics and medical tumors are prospected.

## 2. Fluorescent Polymer Functionalization Strategy

Ideal bioluminescent imaging probes, in addition to high fluorescence efficiency and stable luminescent properties, also need to have good monodispersity in aqueous systems. They have low toxicity to biological organisms and active groups on the surface to facilitate the connection of targeting molecules, achieving the effect of targeted imaging [24,25,26]. However, quantum dots of inorganic functional fluorescent materials, rare earth luminescent materials and noble metal nanoclusters, and semiconducting polymers of organic fluorescent functional materials and organic small molecule fluorescent materials have poor compatibility, poor degradability and long-term biological toxicity in vivo, and other defects. These problems severely limit their application in the biological field [27,28].

**Figure 1:**
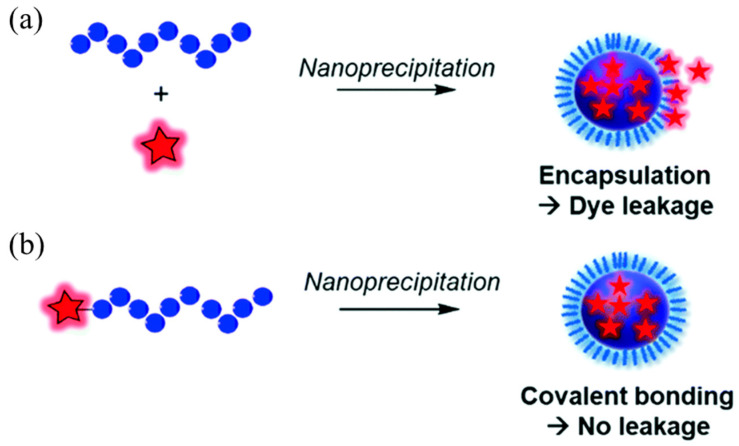
Schematic diagram of the preparation of fluorescent polymer nanoparticles: (**a**) physical encapsulation; (**b**) covalent attachment [31]. Copyright (2021), with permission from Royal Society of Chemistry.

Fluorescent polymer nanomaterials are prepared by combining amphiphilic polymers and fluorescent functional materials using physical doping or covalent linkage. They not only have the characteristics of good optical stability and wide application range of fluorescent functional materials but can also be polymerized by selecting different functional monomers [29,30]. While retaining the good water solubility and excellent biocompatibility of polymer nanomaterials, the controllable synthesis of properties such as size, shape, stability, and surface properties can be achieved. As shown in Figure 1, the currently commonly used strategies for functional modification of fluorescent polymers mainly include physical encapsulation and covalent linkage [31,32,33].

### 2.1. Physical Package

**Figure 2:**
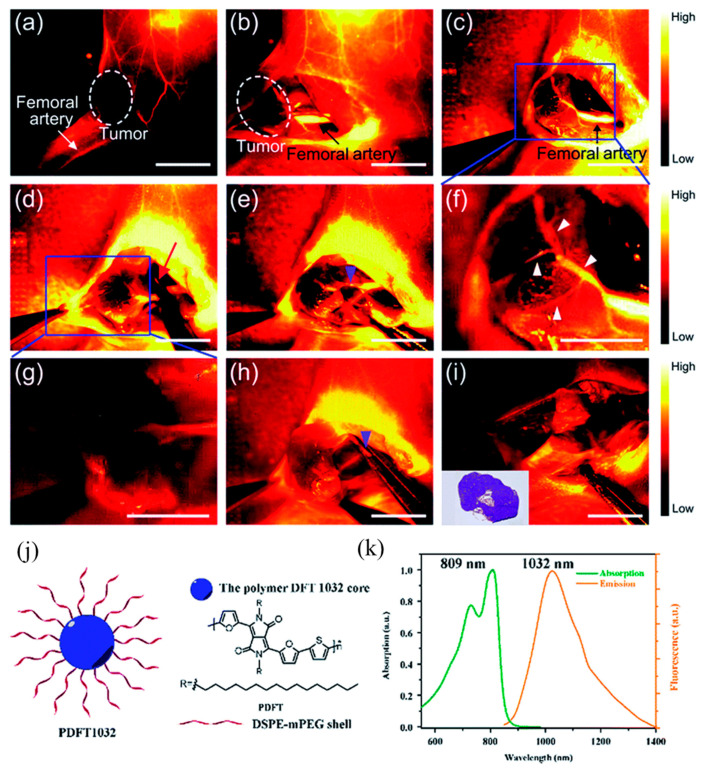
Schematic of DPP-based semiconducting polymer PDFT nanoscale self-assembly and imaging. (**a**) The vascular mapping and the hemodynamic status of the tumor and the femoral artery were determined. The white dashed circle contours the location of the tumor. (**b**,**c**) The branch of the femoral artery that supports the tumor and the vascular network of the tumor (exhibited as a claw shape) were clearly identified. (**d**) A vessel clamp was used to block the blood flow (red arrow) and the signal of the vascular network vanished. (**e**) After 5 min, the clamp was removed and the blood flow of the tumor was still devoid because a temporary thrombus was formed (blue arrowhead). (**f**) Magnification of (**c**). The vascular network of the tumor was clearly identified (white arrowheads). (**g**) Magnification of (**d**). (**h**) The major artery was surgically incised (blue arrowhead). (**i**) NIR-II imaging exhibited the absence of the residual tumor fluorescence and normal circulation (femoral artery) was successfully maintained. Inset is the histological analysis of the osteosarcoma. Scale bar: 8 mm. (**j**) Schematic drawing of a PDFT1032 nanoparticle composed of semiconducting polymer DFT and a hydrophilic DSPE-mPEG shell. (**k**) Absorbance and fluorescence spectrum of PDFT1032 showing an absorption peak at 809 nm and a fluorescence peak at 1032 nm with an 808 nm excitation laser [37]. Copyright (2018), with permission from Royal Society of Chemistry.

Physical encapsulation is the most commonly used method at present. Fluorescent functional materials are directly embedded in polymer nanoparticles using physical encapsulation to prepare fluorescent polymer nanoparticles. The nanoparticles belong to a typical core–shell structure, in which the hydrophilic polymer acts as a protective layer in the shell, and the hydrophobic fluorescent material acts as a fluorescent chromophore in the core. It not only retains the luminescent properties of fluorescent materials, but also improves the stability and biocompatibility of hydrophobic fluorescent materials in water systems [34,35,36]. Yu et al. [37] designed and synthesized a semiconducting polymer PDFT based on diketopyrrolopyrrole (DPP). As shown in Figure 2, the amphiphilic distearoylphosphatidylethanolamine-polyethylene glycol (DSPE-mPEG) was used for physical encapsulation. Self-assembled into an NIR-II fluorescent nanoprobe PDFT1032 with a particle size of 68 nm, the maximum emission wavelength is 1032 nm, and it has excellent photostability, excellent biocompatibility, and extremely low in vivo toxicity. It presents high-resolution, real-time imaging in tumor diagnosis and vascular thrombosis treatment and, more importantly, realizes precise fluorescence imaging “navigation” for in situ tumor surgery and sentinel lymph node biopsy.

If the fluorescent material has hydrophobic properties, the preparation method of physical encapsulation can be used, which has good universality. However, due to the absence of chemical bonds between the polymer and the fluorescent-emitting group, there are situations where the fluorescent material leaks from the fluorescent polymer composite system or the aggregation and quenching of the local fluorescent material occurs, resulting in a decrease in luminescent performance [38,39]. Therefore, how to improve the preparation of fluorescent polymer nanoparticles with stable luminescence using physical encapsulation is still a research hotspot.

### 2.2. Covalent Linkage

**Figure 3:**
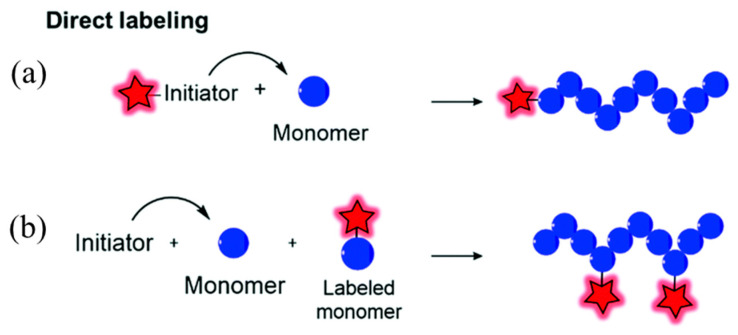
Two forms of covalent linkage: (**a**) polymerization followed by linkage; (**b**) linkage followed by aggregation [31]. Copyright (2021), with permission from Royal Society of Chemistry.

There are two ways to prepare covalently linked fluorescent polymer nanomaterials [31,40]: one is to first copolymerize polymer monomers into polymer chains, and then use covalently linked methods. The fluorescent-emitting group is attached to the polymer chain, and then prepared into nanoparticles. As shown in Figure 3a, it is referred to as “aggregate first and then join”. The nanoparticles prepared in this way have good stability, and the desired multifunctional nanoparticles can be customized by selecting different polymer nanoparticles. However, this preparation method requires functional group matching between empty polymer nanoparticles and fluorescent groups, and its universality is slightly worse than physical packaging. In addition, fluorescent group materials are also prone to fluorescence quenching on the surface of nanoparticles [41,42]. The second approach is to prepare fluorescent groups and polymer monomers into fluorescent polymer monomers, then copolymerize and self-assemble them into fluorescent polymer nanomaterials, as shown in Figure 3b, referred to as “connection first and then polymerization”. The distribution of light-emitting groups in the fluorescent nanoparticles prepared using this method is relatively more uniform, and the optical stability is good. However, there is also the problem that the size of fluorescent polymer nanoparticles is not easy to control due to the steric hindrance of the luminescent group [43,44].

In summary, although the method of physical packaging is used to prepare fluorescent polymer nanomaterials, the distribution of fluorescent materials in nanomaterials is uneven and leaks easily. However, it is still the most commonly used method at present. In order to solve the above problems, designing and synthesizing new fluorescent polymer nanomaterials by means of covalent linkage is still one of the hotspots of scientific research.

## 3. Preparation Method of Fluorescent Polymer Nanomaterials

### 3.1. Active/Controllable Synthesis of Amphiphilic Block Polymers

From the preparation strategy of fluorescent polymer nanomaterials in the previous section, it can be seen that the controllable synthesis of amphiphilic block polymers directly determines the monodispersity, morphology, and size of bioluminescent probes in aqueous solution. At present, the most commonly used living/controllable polymerization methods mainly include atom transfer radical polymerization (ATRP) [45,46] and reversible addition–fragmentation chain transfer polymerization (RAFT) [47]. Below, we focus on the introduction of these two technologies, as shown in Table 1.

#### 3.1.1. ATRP

Matyjaszewski et al. [48] and Sawamoto et al. [49] successively proposed the method of atom transfer radical polymerization (ATRP). The low-valence metal complex M_t_^n^ takes an electron from the initiator organic halide R-X to form R free radicals to initiate monomer aggregation. The formation of chain free radicals P can also take the halogen atom X from the high-valence metal halide M_t_^n+1^-X passivation to P-X, and reduce the high-valence metal halide to M_t_^n^. The reversible transfer equilibrium reaction between the free radical active seeds and the halide dormant seeds of the polymer chains enables effective control of the reactions. Compared with the traditional free radical polymerization, the ATRP method has a wide range of adaptability, can control the molecular weight distribution (PDI) of the polymer between 1.05 and 1.5, and has a mild reaction temperature, simple operation, and is easy to industrialize [50,51,52]. However, when applied to the synthesis of bioluminescent probes, copper-based catalysts have high biotoxicity, and finding other catalysts to replace copper-based catalysts is still a hotspot in ATRP research [53].

#### 3.1.2. RAFT

Compared with the ATRP reaction, the reversible addition–fragmentation chain transfer polymerization (RAFT) reaction system does not involve the participation of copper-based and other biologically toxic transition metals. Additionally, the source of free radicals is basically from the decomposition of organic initiators. For example, azobisisobutyronitrile (AIBN) or dibenzoyl peroxide (BPO) are more suitable for the controlled synthesis of amphiphilic block polymers for biological use [54,55].

The RAFT method was proposed by Rizzardo [56]. The first is initiation (initiation), where the initiator generates free radicals I, then monomers M are initiated to polymerize with each other to generate extended chain free radicals Pn. The second step is the chain transfer reaction (chain transfer); the extended chain free radical Pn reacts with the dithioester chain transfer agent (1) to form an unstable intermediate (2). The groups on both sides of the intermediate can be broken to form a temporarily inactive thioester dormant (3) and a new free radical R·. The third step is to reinitiate the polymerization between the new free radical R· and the monomer to form Pm· (re-initiation). The fourth step is the process of chain equilibrium (chain equilibration), and the macromolecular chain transfer agent (macro-CTA) plays a controlling role. The free radical concentration is low throughout the reaction. Therefore, the molecular weight distribution of the polymer is relatively uniform. The final termination reaction (termination) generally quenches the reaction directly at low temperature, and the product is a mainly macromolecular chain transfer [57,58].

The RAFT mechanism is applicable to a wide range of monomers. The reaction temperature is 60–70 °C, and it has good polymerization ability for monomers such as acrylic acid (AA), methacrylic acid (MAA), and methyl methacrylate (MMA). The resulting polymers are of uniform molecular weight (PDI typically below 1.3). However, this reaction relies heavily on expensive RAFT reagents, and the development of stable, low-cost, and easy-to-synthesize RAFT reagents that meet different systems is still one of the research hotspots [59,60,61].

### 3.2. Preparation Method of Physically Encapsulating Fluorescent Polymer Nanoparticles

**Figure 4:**
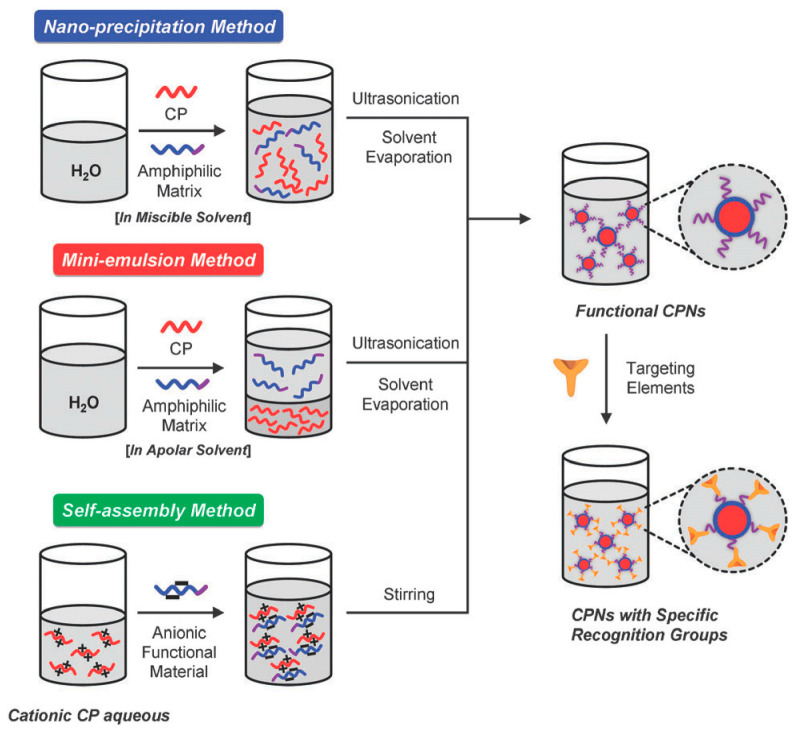
Preparation method for semiconducting polymer nanoparticles [63]. Copyright (2013), with permission from Royal Society of Chemistry.

From the perspective of preparation strategy, fluorescent polymer nanoparticles wrap luminescent materials into amphiphilic block polymers using physical doping. Nanoprecipitation, microemulsion, and self-assembly methods are commonly used, which are similar to the preparation methods of semiconducting polymer nanomaterials, as shown in Figure 4 [62,63].

Nanoprecipitation is a method based on the interfacial deposition of polymers. It was first proposed by Masuhara et al. [64] and then improved by McNeill [65] and Chiu [66]. It is widely used in the preparation of fluorescent nanoparticles in the biological field. Firstly, the fluorescent material and the amphiphilic block polymer material are dissolved in a small amount of good solvent. The material is quickly dropped into a poor solvent (usually deionized water) with vigorous stirring or ultrasound; the huge difference in solubility of the two solvents promotes the aggregation of polymer materials to form nanoparticles, with a particle size of about 15 nm. The process of nanoparticle formation mainly includes several steps: supersaturation, nucleation, coagulation growth, and formation of polymer nanoparticles. The method is simple, fast, and highly reproducible. The nanoparticle colloid has good dispersion and is easy to be functionalized. It is a common method for preparing drug-loaded nanomaterials, but there are also defects such as fewer types of suitable polymers and difficult control of the particle growth process [67].

The microemulsion method is similar to the nanoprecipitation method. The prepared amphiphilic polymer and fluorescent material are first dissolved in a good solvent and then mixed with a poor solvent (usually deionized water) [68]. The huge difference in solubility is used to prepare nanoparticles, but the difference is that a certain concentration of surfactant needs to be added. However, the final surfactant is difficult to remove from the reaction system, which affects the application of fluorescent nanoparticles in the biological field. Additionally, the size of the prepared nanoparticles is relatively large, between 240 and 270 nm [69,70,71].

The usual preparation of the self-assembly method is to dissolve fluorescent materials and functional materials with opposite charges in an aqueous solution according to a certain ratio. After fully stirring and mixing evenly, the functionalized polymer nanoparticles are prepared using high-speed centrifugation and the particle size is about 100 nm. However, the preparation of the self-assembly method also has the instability of nanoparticles in a physiological environment, which limits its further application [72,73].

### 3.3. Preparation Method of Covalently Linked Fluorescent Polymer Nanoparticles

By means of covalent connection, fluorescent materials and amphiphilic block polymer materials are connected and self-assembled into polymer nanoparticles with luminescent properties, mainly including two types of nanoparticles: polymer micelles and nanogels.

#### 3.3.1. Preparation of Polymer Micelles Using Aggregation-Induced Self-Assembly (PISA)

The traditional preparation methods of polymer micelles mainly include the solvent induction method, dialysis method, and direct dissolution method [74,75]. Nanoparticles with morphologies such as spherical, worm-like, and vesicular are prepared using the self-assembly of amphiphilic block polymers with different solubility differences in different solvents. However, the operation is complicated, the reaction takes a long time, and the concentration of the prepared nanoparticles is low (≤1 mg/mL), which makes it impossible to achieve large-scale mass production [76,77,78]. In recent years, the polymerization-induced self-assembly (PISA) method can not only prepare micelles and assemblies with different morphological structures (including spherical, worm-like, vesicular, etc.) in one pot, but nanoparticles with solids content up to 50% can also be synthesized in bulk [79,80]. This provides a new idea for the commercial application of preparing polymer nanoparticles, and is also widely used in the fields of drug-controlled release, bioimaging, and catalysis [81,82].

**Figure 5:**
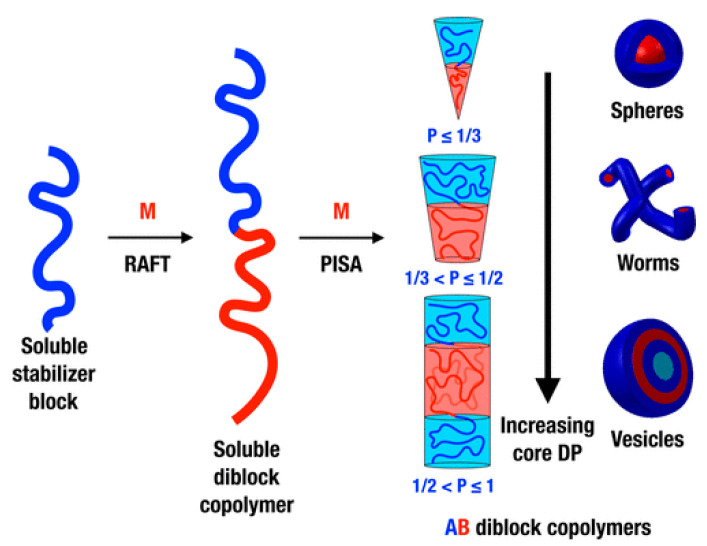
Schematic diagram of the preparation of nanoparticles from diblock polymers using the PISA method [86]. Copyright (2016), with permission from American Chemical Society.

Hawkett et al. [83] first used polyacrylic acid (PAA) as a water-soluble macromolecular RAFT chain transfer agent and induced self-assembly into spherical micelles in aqueous solution. Subsequently, Pan et al. [84,85] utilized poly-4-vinylpyridine (P4VP) as a macromolecular RAFT chain transfer agent for dispersion polymerization in methanol solvent. With the chain growth of PS spheres, the morphology of the polymer gradually changed from spherical to worm-like and vesicle-like. A schematic diagram of nanomaterials prepared using the aggregation-induced self-assembly method [86] is shown in Figure 5. The water-soluble polymer chain transfer agent (macro-CTA) prepared using the RAFT method initiates another hydrophobic polymer monomer, and the newly synthesized diblock polymer can be dissolved in the reaction system at the early stage of the reaction. With the continuous growth of the second hydrophobic chain, the volume of the insoluble polymer continues to increase. When the critical micelle concentration (CMC) is reached, it self-assembles into different morphologies. A series of theoretical studies have shown that the morphology of block polymer self-assembly is determined by the volume ratio P of the polymer at the hydrophobic end. When P ≤ 1/3, the diblock polymer exhibits spherical nanoparticles. When 1/3 < P ≤ 1/2, the diblock polymer exhibits worm-shaped nanoparticles. When 1/2 < P ≤1, the diblock polymer exhibits a vesicle shape.

#### 3.3.2. Preparation of Nanogel Polymer Microspheres Using Precipitation Polymerization

Hydrogel is a kind of hydrophilic polymer material with a three-dimensional network structure, which can absorb water several times the weight of the material and has good biocompatibility and degradability [87]. Nanohydrogels are hydrogels with a size between 100 and 1000 nm that have the dual characteristics of hydrogels and nanomaterials. The structure is stable under physiological conditions, and it has a high drug loading rate and a long drug release cycle. It is a drug carrier material that has developed rapidly in recent years [88,89].

**Figure 6:**
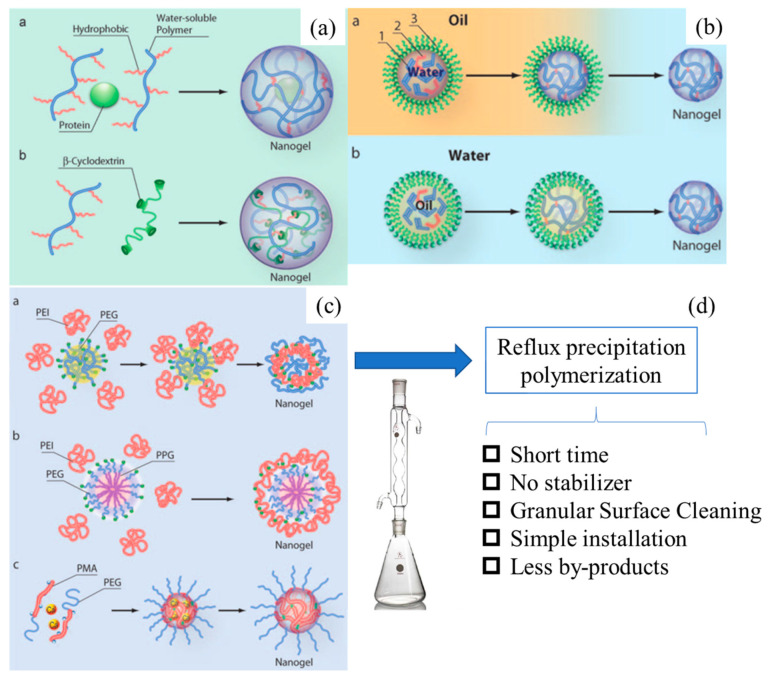
Schematic diagram of the nanohydrogel preparation method: (**a**) polymer self-assembly method; (**b**) inverse emulsion polymerization method; (**c**) precipitation polymerization method; (**d**) reflux precipitation polymerization method [90]. Copyright (2009), with permission from Wiley.

The nanogel preparation method [90] is shown in Figure 6. The traditional preparation methods mainly include emulsion polymerization, microemulsion polymerization and dispersion polymerization, all of which need to add stabilizers or surfactants to stabilize the reaction system and avoid aggregation and precipitation. However, it is difficult to remove the stabilizer or surfactant from the reaction system after the reaction, which affects the application of polymer microspheres in the biomedical field [91]. The method of precipitation polymerization was first proposed by Chibante et al. [92]. The stabilizer is replaced by a cross-linking agent, which is added to the reaction system together with the reactive monomer, and polymer microspheres with uniform particle size and clean surface are prepared after heating and polymerization. However, this method is only suitable for the polymerization of hydrophobic monomers and cannot prepare polymer microspheres for biomedicine. To expand the application of precipitation polymerization to hydrophilic monomers, Yang et al. [93] developed a distillation precipitation method, which shortened the precipitation polymerization time to 1–2 h. However, with the decrease in the solvent amount in the reaction system, the late reaction was unstable and the product yield was low. Wang et al. [94] developed the reflux precipitation polymerization method based on the technique of distillation precipitation. A return-shaped condenser was connected to the reaction device to ensure that the volume of the solvent in the reaction system remained unchanged, making the polymerization of the reaction system more stable. Compared with the traditional preparation method, the reflux precipitation polymerization method has the characteristics of short time consumption, no need for stabilizer, clean particle surface, simplicity of device, and less byproducts.

## 4. Biotoxicity of Fluorescent Polymer Nanomaterials

Due to their unique physical and chemical properties, nanomaterials have broad application prospects in the field of biomedicine. For example, as a drug carrier or a bioimaging probe, the pharmacokinetics and potential toxic effects in the organism need to be tested before practical application. In vivo toxicity studies of nanomaterials involve a variety of exposure methods, such as intravenous, transdermal absorption, subcutaneous, inhalation, intraperitoneal, and oral administration, and various animal models such as mice, rats, dogs, and monkeys. After nanomaterials enter the body and interact with biological components (proteins, cells), they are distributed to different organs of the body. At this point, the particles maintain their original structure or degrade. The slow removal and accumulation of materials, as well as the large number of phagocytes make the liver, spleen, and other organs in the reticuloendothelial system the most important targets of oxidative stress of nanomaterials. In addition, organs with high blood flow, such as lungs and kidneys, are also affected by nanomaterials [95].

Current toxicological mechanisms of nanomaterials mainly focus on the hypothesis of free radical oxidative damage [96]. This hypothesis holds that, under normal conditions, the content of reactive oxygen species in the mitochondria of body cells is very low. Additionally, there are many antioxidant systems in the body, and the active oxygen free radicals produced by normal cell metabolism are easily removed by glutathione reductase and antioxidant enzymes. When nanoparticles enter the body, they can induce the production of a large amount of reactive oxygen species (ROS). ROS mainly activate the inflammatory response by activating the phosphorylation of NF-κB transcription factors and MAPKs. As a result, the antioxidant defense system in the mitochondria is destroyed, causing various damages and further affecting the normal physiological functions of the body.

Li et al. [97] found that nanoparticles can induce an increase in reactive oxygen species in RAW264.7 cells, leading to cell apoptosis. Shvedova et al. [98] summarized the main mechanism of nanoparticle-induced ROS generation in cells, resulting in oxidative damage: (1) oxidation of liposomes in mitochondria; (2) NADPH oxidation leading to cell apoptosis and inflammatory response; (3) depletion of reduced glutathione in the body; (4) activation of peroxidase, leading to degradation of nanoparticles. At the same time, this is precisely the oxidative damage effect of nanoparticles exposed to the body.

In addition to ROS, reactive nitrogen species (RNS) may also be involved in the free radical oxidative damage effect of nanoparticles. Recent studies have proved that RNS play a role in the inflammatory damage caused by nanoparticles. Lanone and Boczkowski suggested that the main molecular mechanism of in vivo toxicity of nanomaterials is the induction of free radicals leading to oxidative damage [99]. Free radicals can not only cause damage to biological components by oxidizing lipids, proteins, and DNA, but also induce and enhance inflammation by upregulating redox-sensitive transcription factors (such as NF-kB) and inflammation-related kinases [100,101]. The composition of some materials, such as iron, cadmium, chromium, and other atoms also affects the toxicity in vivo. In addition, surface modification of nanoparticles can alter their interaction with cell membranes, resulting in their altered cellular uptake, thereby affecting their toxicological effects on targeted cells. The application and toxicity of different fluorescent nanomaterials in biological systems are shown in Table 2.

Because of the small size effect of nanomaterials and the complexity of biological systems, the effects caused by the processing of nanomaterials in biological systems are unpredictable. The interaction between biological components (proteins and cells) and nanostructured materials may cause unique biodistribution and metabolic reactions, making it difficult to predict the metabolism and safety of nanomaterials in biological systems. As evidenced by the above literature review, fluorescent materials have been widely used in biomedical fields such as bioimaging, biosensors, and drug delivery because of their excellent luminescent properties and photoconversion properties. However, due to their particularity, their biological safety cannot be predicted. Therefore, they may cause certain harm to organisms during use, which greatly limits the application of fluorescent nanomaterials. Therefore, it is urgent to find fluorescent nanomaterials with good biocompatibility.

## 5. Application of Fluorescent Polymer Nanomaterials in Bioimaging

In order to meet the needs of different biological applications, many different types of fluorescent polymer nanomaterials have been developed. This paper focuses on the research direction and focuses on the introduction of polymer nanomaterials based on rare earth luminescent materials, semiconducting polymers, and organic, small molecule luminescent materials [7,8,9]. The use of nano-fluorescent probes can quickly, accurately, and selectively label and study target molecules on cells. Labels were obtained according to their excitation wavelengths at different emission wavelengths, as shown in Table 3.

### 5.1. Polymer Nanomaterials Based on Rare Earth Luminescent Materials

Rare earth elements include lanthanides with atomic numbers 57–71 in the periodic table of chemical elements and scandium (Sc, 21) and yttrium (Y, 39) with similar chemical properties, a total of 17 elements [102]. The electron configuration of lanthanides is [Xe]4f^0–14^5d^0–16^s^2^, and each element has a 4f electron shell. Compared with other luminescent materials, it has narrow emission band (10–20 nm), high luminous efficiency, large Stokes shift, and long emission lifetime (range μs-ms) features [103]. At present, rare earth luminescent materials used in biological imaging mainly include rare earth organic complexes and rare earth upconversion materials.

#### 5.1.1. Rare Earth Organic Complexes

**Figure 7:**
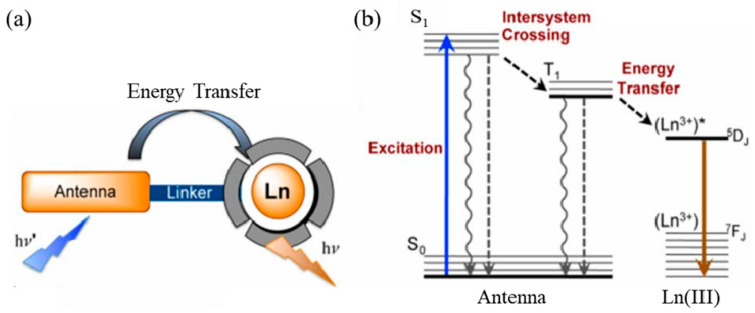
(**a**) Schematic diagram of the antenna effect of rare earth complexes; (**b**) schematic diagram of the luminescent principle of rare earth complexes [103]. Copyright (2014), with permission from American Chemical Society.

Rare earth organic complexes are due to the small molar absorptivity of lanthanide trivalent ions and the prohibition of ff transitions in the electron shell [104,105]. As a result, very little energy is directly absorbed by the 4f energy level of lanthanide elements, and organic ligands are required to act as antennas to absorb excitation energy, thereby sensitizing rare earth ions to emit light. This process is called “antenna” (Figure 7a) [106]. As shown in Figure 7b, the main process of energy transfer of rare earth complexes is as follows: (1) After the rare earth complex absorbs energy, electrons transition from the ground state (S0) to the excited state (S1). (2) The energy of the excited state (S1) transfers energy to the lowest excited triplet state (T1) through intersystem crossing (ISC). (3) When the lowest excited triplet state (T1) matches the lowest excited state energy level (5DJ) of rare earth ions, energy transfer occurs between T1 and 5DJ. Eventually, the rare earth ions return to the ground state (7DJ) in the form of radiation, thereby emitting the characteristic fluorescence of rare earth ions [103,107,108].

According to the structure of the ligands, it can be divided into β-diketone ligands, carboxylic acid ligands and macrocyclic ligands such as crown ethers [109]. There are coordination elements such as O, N, and S on the ligand, and a stable six-membered ring structure is formed after coordination with rare earth. It can directly absorb laser energy and effectively transfer energy to rare earth ions through the structure of the six-membered ring, and then emit the characteristic fluorescence of rare earth ions. The coordination ability of the coordinating atoms is O > N > S. When rare earth complexes are used in biological imaging, water molecules easily replace the coordination bonds of organic ligands [110]. The high-frequency O–H bonds of water increase the nonradiative decay of excited states of rare earth ions, which in turn affects the luminescent properties of rare earths [111]. When rare earth organic complexes are used as fluorescent probes, they are usually physically wrapped with biocompatible silica or polyethylene glycol to form a core–shell structure, which improves the chemical stability of the material and avoids the interference of water molecules.

**Figure 8:**
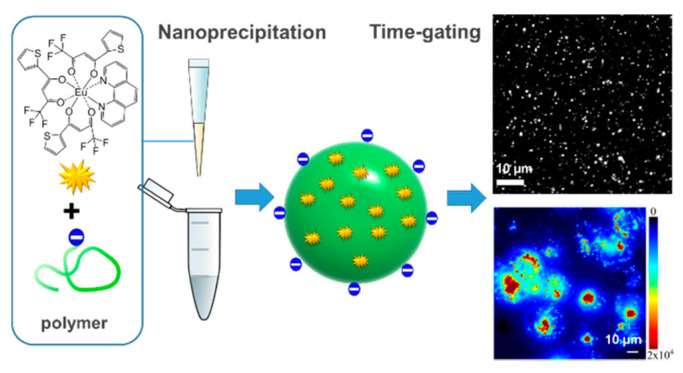
Schematic diagram of rare earth polymer nanoprobes for single particle detection and cell imaging [112]. Copyright (2019), with permission from American Chemical Society.

Dos Santos et al. used the polymer PMMA-COOH to physically wrap the rare earth complex Eu(TTA)_3_phen, and the preparation’s schematic diagram is shown in Figure 8. By adjusting the concentration of the precursor, rare earth polymer nanoparticles of 10 nm, 20 nm, and 30 nm were prepared. The fluorescence quantum efficiency exceeded 20%, and the brightness of a single particle was as high as 4.0 × 10^7^ M^−1^ cm^−1^. A lower laser intensity of 0.24 W/cm^2^ can be used to image single particles, and time-resolved imaging microscopy can be used to dynamically observe the progress of nanoparticles into cells [112]. However, the polymer physically wraps the rare earth complex material, and there is also uneven distribution of the fluorescent material. Aggregation quenching is prone to occur, which affects the luminescent properties of fluorescent probes, and the related preparation methods still need further research [113,114,115].

**Figure 9:**
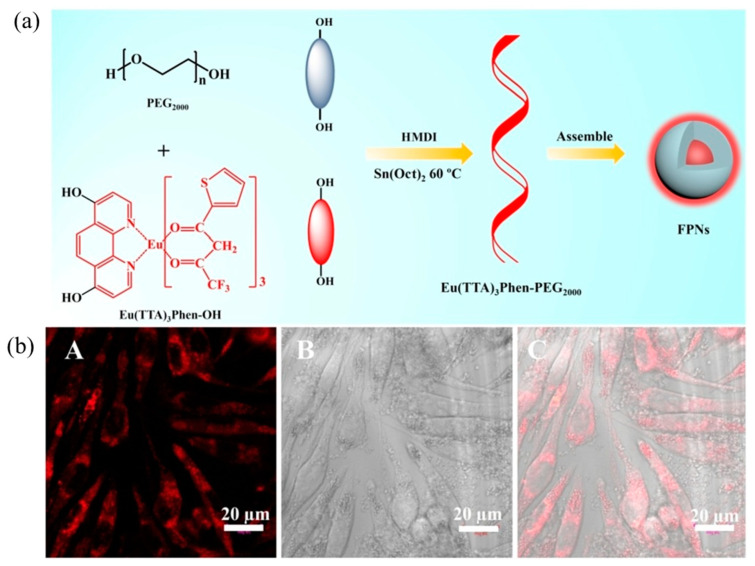
[Eu(TTA)3phen]-PEG2000 nanomaterials: (**a**) schematic diagram of preparation; (**b**) cell imaging diagram. (**A**) fluorescent image 405 nm laser excitation, (**B**) bright field and (**C**) merged image of (**A**,**B**). Scale bar ¼ 20 mm. [116]. Copyright (2018), with permission from Elsevier.

**Figure 10:**
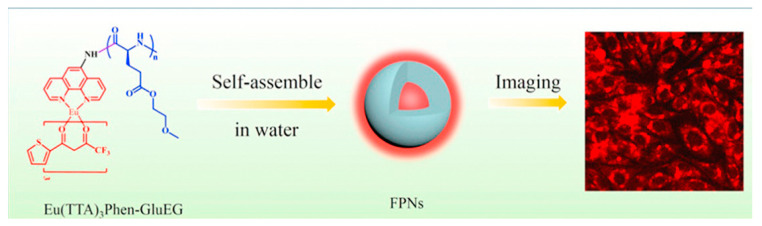
Preparation and cell imaging of [Eu(TTA)_3_phen]-GluEG nanomaterials [117]. Copyright (2018), with permission from Elsevier.

In order to solve the problem of uneven distribution of fluorescent materials in polymer nanosystems, Xu et al. [116,117] modified hydroxyl and amino groups on Eu(TTA)_3_phen, respectively. As shown in Figure 9a and Figure 10, two complexes, [Eu(TTA)_3_phen]-OH and (Eu(TTA)_3_phen]-NH_2_, were prepared. They then reacted with hydrophilic polymers PEG2000 and GluEG NCA through covalent connection to generate [Eu(TTA)_3_phen]-PEG2000 and [Eu(TTA)_3_phen]-GluEG, respectively, and self-assembled into water-soluble nanoparticles. They emi the 614 nm characteristic peak of Eu(III) ion in aqueous solution, and can be successfully taken up by L929 cells and HeLa cells, and emit strong red light (as shown in Figure 9b and Figure 10).

#### 5.1.2. Rare Earth-Doped Upconversion Luminescent Materials

**Figure 11:**
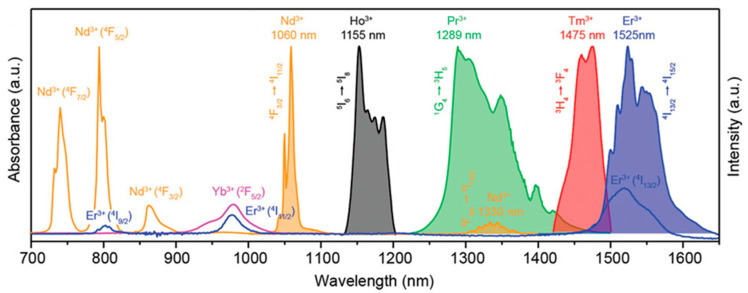
Absorption spectra of Nd^3+^, Er^3+^, and Yb^3+^ in NIR-I and emission spectra of Nd^3+^, Ho^3+^, Pr^3+^, Tm^3+^, and Er^3+^ in NIR-II [118]. Copyright (2019), with permission from Wiley.

Rare earth-doped upconversion materials are doped with trivalent lanthanide ions into a suitable dielectric matrix lattice. The lanthanide ions act as the luminescent center, and the ground state electrons of the sensitized ions are first excited to the excited state under the irradiation of the excitation light of a suitable wavelength. Then, the energy is transferred to the luminescent center, and the luminescent center is excited to an excited state. Finally, the excited state electrons return to the ground state and emit near-infrared fluorescence. Therefore, sensitizing ions are required to have a larger absorption cross-section at NIR-I or NIR-II. For example, the luminescent central ions with NIR-I emission include Nd^3+^, Yb^3+^, and Er^3+^, and the luminescent central ions with NIR-II emission include Nd^3+^, Ho^3+^, Pr^3+^, Tm^3+^, and Er^3+^ (as shown in Figure 11) [118]. Rare-earth-doped upconversion materials have the advantages of low toxicity, narrow-band emission, long emission lifetime, no photobleaching, and no scintillation. They have broad application prospects in the fields of bioimaging, such as in analytical sensors, PDT, and optical imaging [119].

**Figure 12:**
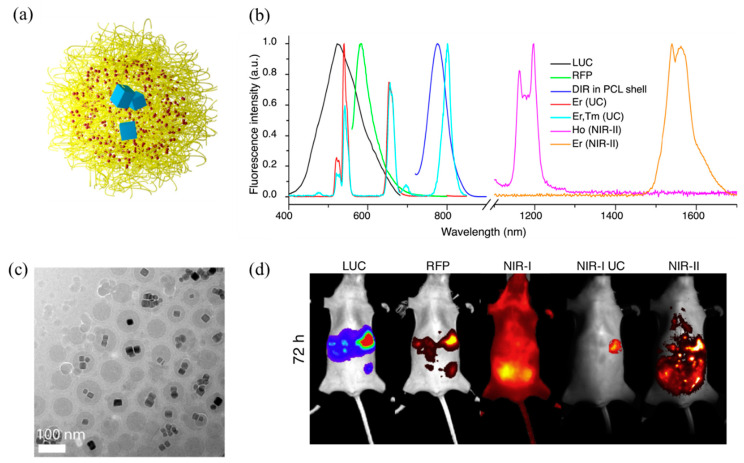
(**a**) Schematic diagram of nanoparticles, in which the yellow block represents the block polymer PEO-b-PCL, the red point represents the NIR-I quantum dot DiR, and the blue block represents the NIR-II quantum dot LNPs; (**b**) the nanoparticle TEM image; (**c**) fluorescence spectrum of multifunctional nanomaterials; (**d**) imaging of multifunctional nanoparticles in mice [120]. Copyright (2017), with permission from Elsevier.

Rare earth-doped upconversion luminescent materials are generally hydrophobic. Generally, water-soluble bioluminescent probes are prepared by modifying the surface function of materials with SiO_2_ that has better biocompatibility, water-soluble polymers (PEG, PAA, PEI, etc.), and polyols. Tao et al. [120] used the block polymer PEO-b-PCL to wrap NIR-I emitting rare earth nanocrystalline materials NaYF_4_:Yb/Ho(DiR) and NIR-II-emitting NaCeF_4_:Er/Yb(LNPs). And luciferase (LUS) and red fluorescent protein (RFP) were doped into the polymer to prepare a multifunctional nanomaterial that can simultaneously generate NIR-I and NIR-II fluorescence spectra (as shown in Figure 12a–c). Then, the nanomaterials were injected intraperitoneally into mice with ovarian cancer, and fluorescence signals of two spectra could be found in the ovarian LUS+/RFP+-responsive cancer cell OVCAR-8 (Figure 12d).

### 5.2. Polymer Nanomaterials Based on Semiconducting Polymers

**Figure 13:**
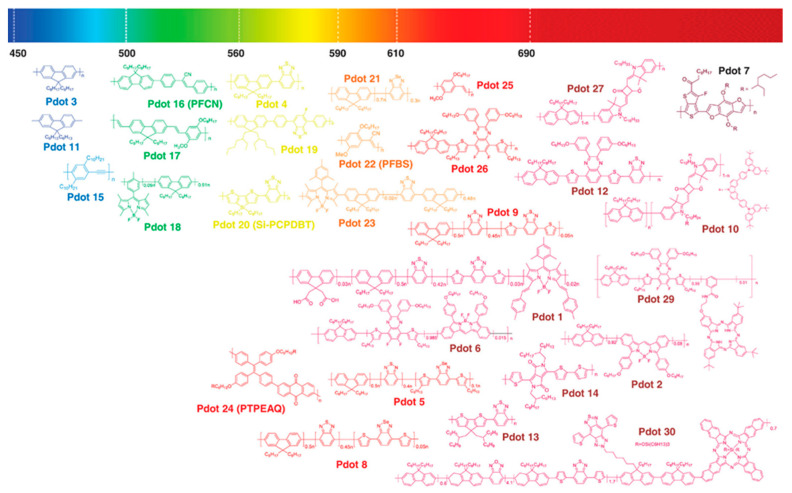
Chemical structures of semiconducting polymers [125]. Copyright (2019), with permission from Wiley.

Semiconductor polymers (SPs) are a class of polymer materials whose main chain is composed of π–π-conjugated structures [121,122]. Due to the excellent optoelectronic properties and good processing properties of semiconductors, they were first applied in the field of organic optoelectronics. At present, semiconducting polymer materials commonly used in the biomedical field are divided into polyfluorene (PF), polythiophene (PT), poly(phenylene ethylene, PPE) and poly(p-phenylene vinylene, PPV) according to the structure of the main chain. Their derivatives [123,124] and structure are shown in Figure 13.

From the perspective of the main chain structure of the semiconducting polymer, it is a typical rigid structure and hydrophobic, and aggregation occurs in aqueous solution, resulting in fluorescence quenching. At present, the commonly used method is to directly physically encapsulate semiconducting polymer materials in amphiphilic block polymers to prepare water-soluble polymer fluorescent nanoparticles (SPNs). Not only can this effectively solve the problem of monodispersion of semiconducting polymers in water, but the surface can also be easily functionalized. They have been widely used in the fields of tumor diagnosis [126,127,128] and antibacterials [129,130,131]. Among them [15], polyethylene glycol (PEG) is widely used to modify semiconducting polymers because of its characteristics of increasing drug solubility, reducing body immunity, and prolonging the residence time of drugs in the body [132].

**Figure 14:**
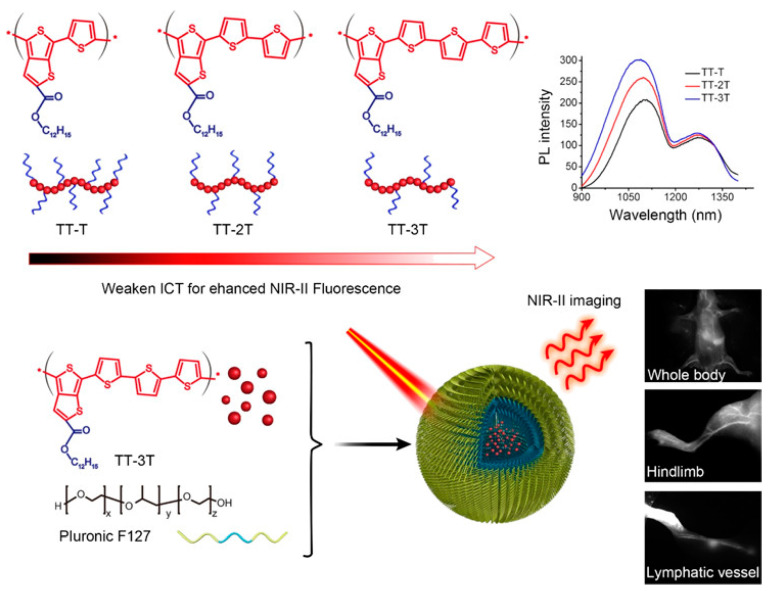
Schematic diagram of the synthesis of ester-based semiconducting polymer TT-3T CPs and their application in bioimaging [133]. Copyright (2019), with permission from American Chemical Society.

In order to improve the fluorescence quantum efficiency of semiconducting polymers, Fan et al. used low-energy-band ester-based semiconducting polymers to skillfully control intramolecular charge transfer (ICT) to increase the intensity of NIR-II fluorescence [133]. As shown in Figure 14, as the thiophene group chain lengthened (TT-T to TT-3T), the ICT gradually weakened, and the corresponding NIR-II fluorescence emission gradually increased. TT-3T CPs (51–70 nm) were prepared by physically wrapping TT-3T with amphiphilic block polymer F127. They emit NIR-II light (1050 nm) in aqueous solution with a fluorescence quantum efficiency of 1.75%. Moreover, in vivo cell tracking, vascular system imaging, and lymphatic drainage mapping all had good imaging effects and high NIR-II spatial resolution.

**Figure 15:**
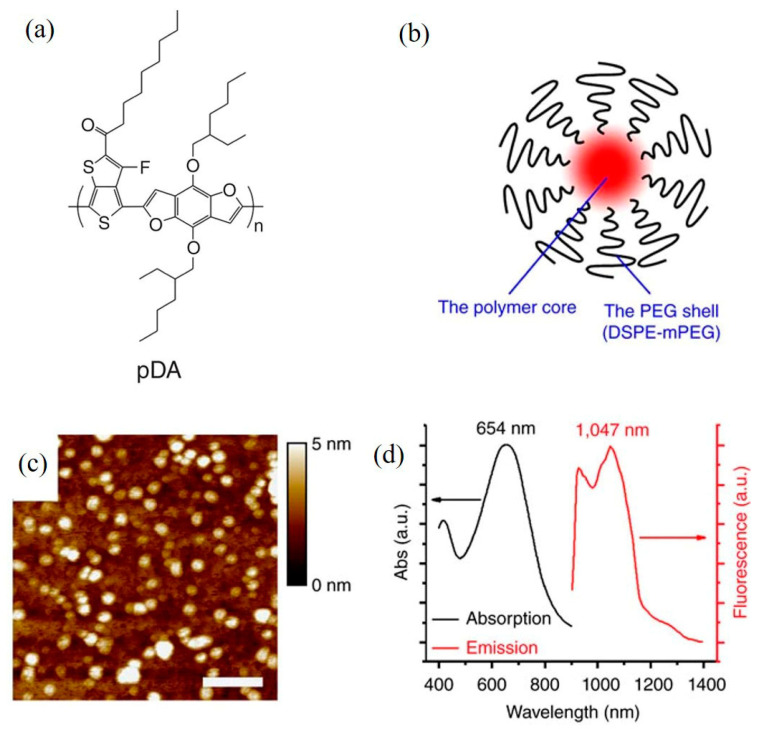
(**a**) Molecular structure diagram of semiconducting polymer pDA; (**b**) schematic diagram of pDA-PEG nanoparticles; (**c**) AFM image of pDA-PEG nanoparticles; (**d**) absorption and emission diagram of pDA-PEG nanoparticles aqueous solution [134]. Copyright (2014), with permission from Springer Nature.

In order to further study the NIR-II real-time imaging application of semiconducting polymers in vivo, Hong et al. [134] designed and synthesized a NIR-II semiconducting polymer pDA (Figure 15a). After being physically encapsulated with the amphiphilic block polymer DSPE-MPEG (5kDa), pDA-PEG nanoparticles with a particle size of 2.9 nm were prepared (as shown in Figure 15b,c). The fluorescence emission wavelength is about 1000 nm (Figure 15d), the fluorescence quantum efficiency is about 1.7%, and it has been successfully applied to real-time imaging of vascular diseases.

**Figure 16:**
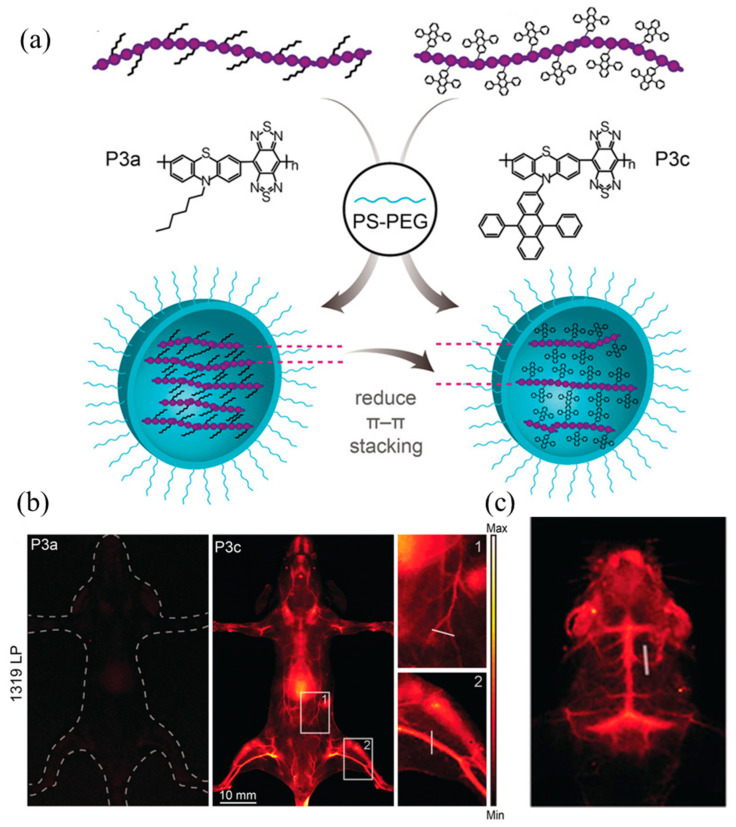
(**a**) Schematic diagram of the preparation of P3a NPs and P3c NPs; (**b**) NIR-II images of mice injected with P3a NPs and P3c NPs, respectively; (**c**) vascular NIR-II images of skull and brain of mice injected with P3c NPs [137]. Copyright (2020), with permission from Wiley.

Some semiconducting polymers are prone to the aggregation-caused quenching (ACQ) phenomenon after they are prepared into nanoparticles using physical encapsulation of amphiphilic block polymers [135,136]. Zhang et al. [137] used phenothiazine, which has typical AIE characteristics, as the electron donor. As shown in Figure 16a, different groups were introduced into the side chains to compare the effect of weakening ACQ. The study found that the emission wavelength of P3c modified with 9,10-diphenylanthracene (9,10-diphenylanthracene) was larger than that of P3a modified with hexane, and the emission intensity was high. Then the polymers P3a and P3c were physically encapsulated using the amphiphilic block polymer PS-PEG to prepare P3a NPs and P3c NPs, which were injected into mice. As shown in Figure 16b,c, the mice in the P3c NPs group glowed red, while the mice in the P3a NPs group did not. Additionally, because P3c NPs have strong NIR-II luminescent properties, the skull and cerebral blood vessels of mice can be clearly observed when performing imaging in mice.

Fluorescence brightness is determined using the absorption cross-section and the fluorescence quantum efficiency. Fluorescence quantum efficiency refers to the ratio of the number of emitted photons to the number of absorbed photons, which is one of the important parameters for evaluating the performance of fluorescent probes. The emission wavelength range of polymer quantum dot PFBT is comparable to that of Qdot 565, a commonly used fluorescent probe, inorganic semiconductor quantum dot, and IgG-Alexa 488, which contains approximately six dye molecules per IgG antibody. Therefore, the photophysical properties of the three are summarized and compared (see Table 4).

It can be seen from Table 1 that, when the three nanoparticles are excited by a laser with a wavelength of 488 nm, the single-particle luminescence brightness of PFBT quantum dots with a size of about 10 nm is about 30 times higher than that of IgG-Alexa 488 and Qdot 565. At a wavelength of 488 nm, the absorption cross-section of PFBT quantum dots is approximately half of its own peak absorption cross-section. The luminescence brightness of the three fluorescent probes was compared in parallel using single-particle imaging experiments [139]. Experiments have found that when the probe is excited by a laser with a wavelength of 488 nm, when the excitation power is low, a single PFBT nanoparticle with high luminescence brightness close to the diffraction limit can be observed. Under the same conditions, the luminescence of IgG-Alexa 488 and Qdot 565 probes was found to be very weak. The camera used in the experiment barely captured the fluorescent signal. After counting the fluorescence intensity distribution of thousands of nanoparticles, it was found that the luminescence brightness of PFBT nanoparticles was about 30 times higher than that of IgG-Alexa 488 and Qdot 565 probes. These experimental data are consistent with the results based on the comparison of photophysical parameters.

### 5.3. Polymer Nanomaterials Based on Organic Fluorescent Small Molecules

**Figure 17:**
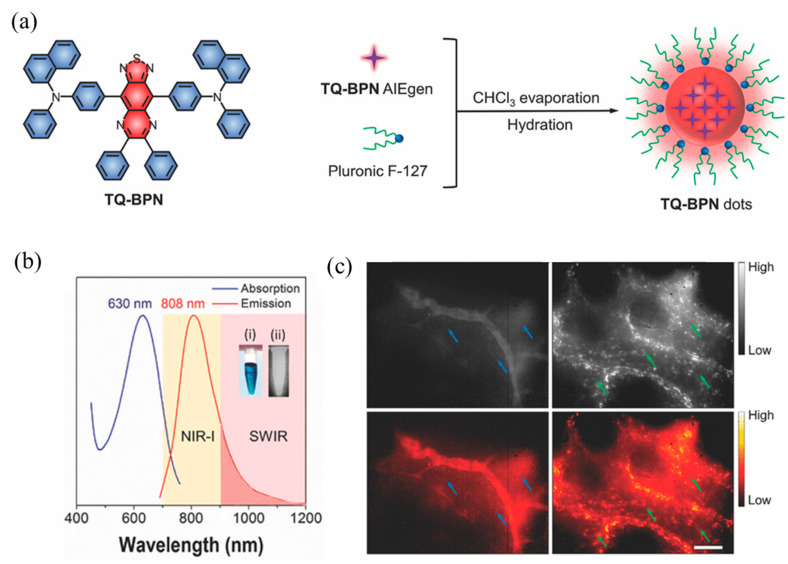
(**a**) Schematic diagram of TQ-BPN dot preparation; (**b**) TQ-BPN dot absorption and generation curves in aqueous dispersion; (**c**) NIR-II of TQ-BPN dots at different stages of tumor growth imaging [143]. Copyright (2018), with permission from Wiley.

**Figure 18:**
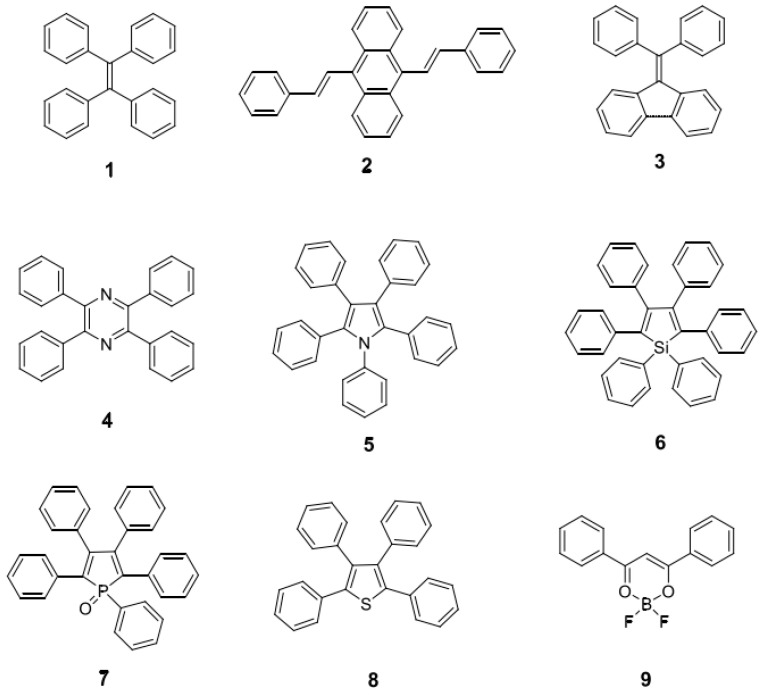
Typical AIE small molecules and their structures. 1. Tetraphenylethene (TPE); 2. Triphenylamine (TPA); 3. Phenothiazine (PTZ); 4. Benzothiazole (BTH); 5. 2-(2′-Hydroxyphenyl)benzoxazole (HBO); 6. 2-(4′-Biphenylyl)-5-(4″-tert-butylphenyl)-1,3,4-oxadiazole (BBD); 7. 1,2,3,4,5-pentaphenylsilole (PPS); 8. 1,2-bis(4′-phenylvinyl)benzene (DPVBi); 9. 1,4-bis(2,2-diphenylvinyl)benzene (DPVBi-Ph).

The luminescence intensity of fluorescent probes determines the signal-to-noise ratio and imaging depth of the probes. If polymer nanoparticles with higher brightness are to be prepared, more fluorescent materials need to be encapsulated into the polymer nanoparticles. However, in most fluorescent probe materials, as the concentration increases, the fluorescence aggregation-induced quenching (ACQ) phenomenon occurs [140,141]. Tang et al. [142] first proposed the aggregation-induced emission (AIE) phenomenon, and AIE materials are an effective way of solving the above problems. Qi et al. [143] designed and synthesized the AIE compound TQ-BPN, and prepared TQ-BPN nanoparticles with a particle size of 33 nm after physical wrapping with the amphiphilic block polymer Pluronic F-127 (as shown in Figure 17a). The fluorescent quantum effect was as high as 13.9%. Although the maximum emission wavelength was in the NIR-I region (808 nm), there was still a fluorescence quantum efficiency of 2.8% in the NIR-II region (Figure 17b). Additionally, when TQ-BPN nanoparticles were used as fluorescent probes to perform fluorescence imaging on a mouse’s brain, it was found that the imaging spatial resolution reached 2.6 μm and the penetration depth reached 150 μm. More importantly, as shown in Figure 17c, clearly identifiable fluorescent signals can be seen at various stages of tumor growth, which can be applied to early diagnosis of cancer. According to the mechanism of aggregation-induced luminescence, more and more AIE molecules have been designed and synthesized by researchers. Typical AIE small molecules include hydrocarbon molecules (1–3 molecules) and heterocyclic small molecules (4–9 molecules) (see Figure 18).

**Figure 19:**
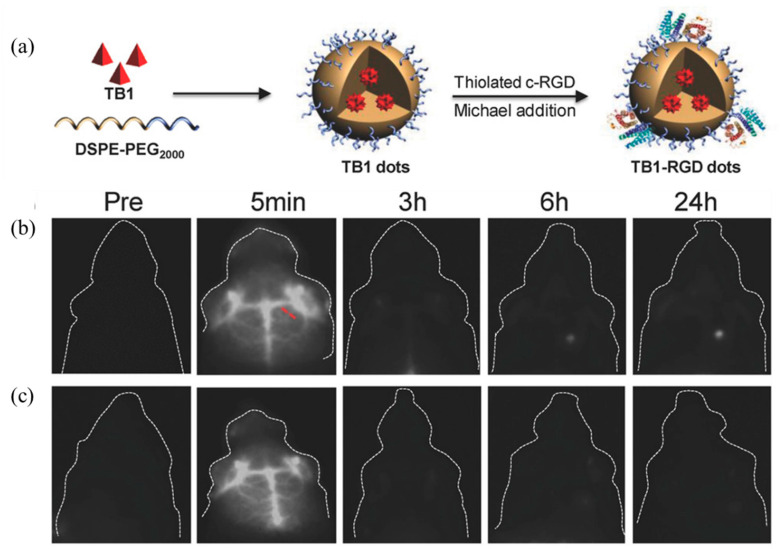
(**a**) Schematic diagram of the preparation of TB-1 dots and TB-RGD dots.; (**b**) imaging of TB-RGD dots injected into mice at different times. The red dashed line indicates the location of the intercepted area for calculating intensity of luminescence; (**c**) images of different time intervals of TB-1 dots injected into mice [144]. Copyright (2018), with permission from Wiley.

In order to further improve the fluorescence efficiency of fluorescent probes in NIR-II, Sheng et al. [144] designed and synthesized a new AIE material, TB-1, containing a DA structure. TB-1 dots with a particle size of 32 nm were prepared after physical encapsulation using DSPE-PEG2000, and the schematic diagram of the preparation is shown in Figure 19a. The maximum emission wavelength of the nanomaterial exceeded 1000 nm in the aqueous dispersion system, and the fluorescence efficiency was as high as 6.2%. The blood vessels in the brain of the mouse could be clearly seen without opening the mouse’s cranium. In addition, the targeting group c-RGD was further modified to the surface of nanoparticles using a Michael addition reaction, prepared into TB1-RGD dots, and then injected into mice, respectively. From the comparison of Figure 19b,c, it can be seen that the TB1-RGD dot group modified with the targeting group has obvious imaging in the tumor part of the mouse at 24 h. The corresponding TB-1 dots with unmodified targeting groups had no obvious imaging effect.

**Figure 20:**
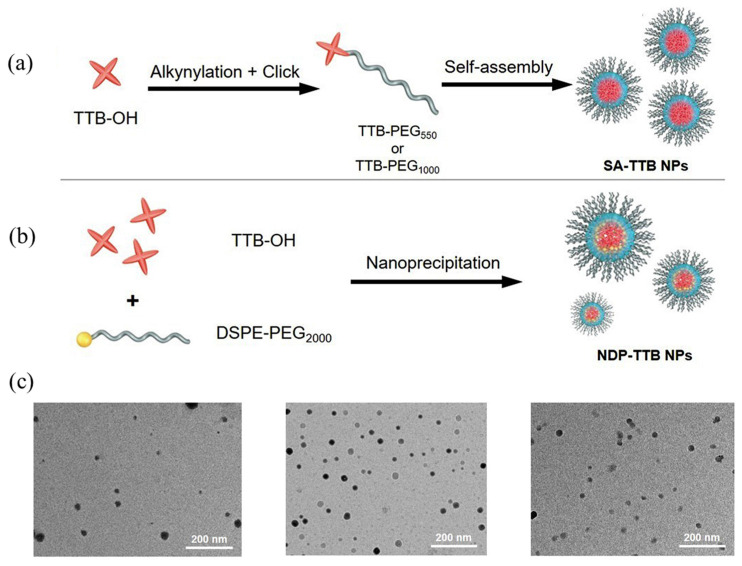
(**a**) Schematic diagram of TTB-OH self-assembly to prepare nanoparticles; (**b**) TTB-OH physical encapsulation method to prepare nanoparticles; (**c**) TEM images of three kinds of nanoparticles [145]. Copyright (2021), with permission from Elsevier.

Although the AIE material is wrapped in the polymer model using physical wrapping, the monodispersity and biocompatibility of the water system of the AIE material improve. However, there are also nanomaterials prepared using physical encapsulation in different batches, showing different particle sizes and encapsulation effects. To prepare AIE nanomaterials with good uniformity, Li et al. [145] alkynylated the AIE material TTB-OH. As shown in Figure 20a, amphiphilic polymers were prepared using amino-alkyne click polymerization with the amino-modified hydrophilic PEG polymer PEG-NH_2_, which then self-assembled into SA-TTB NPs. At the same time, as a comparison, as shown in Figure 20b, NDP-TTB NPs were prepared by directly encapsulating TTB-OH with the amphiphilic block polymer DSPE-PEG2000. From the TEM comparison images of nanoparticles in Figure 20c III, it can be seen that the two SA-TTB NPs prepared using the self-assembly method are more uniform in particle size and better in monodispersity than the NDP-TTB NPs prepared using the physical encapsulation method. By dispersing the three kinds of nanoparticles into the water system, it was measured that their maximum emission wavelengths were all around 1050 nm, with little difference. However, the fluorescence efficiency of SA-TTB NPs in the water system was as high as 10.3%, which is much higher than that of physically encapsulated NDP-TTB NPs. Additionally, a resolution of 38 μm and a penetration depth of 1 cm can be achieved in mice.

In addition, based on the typical donor–acceptor–donor (D–A–D) structure, benzobisthiadiazole (BBTD) derivatives are representative organic small molecules [146,147]. With good biocompatibility, low biotoxicity, easy functionalization, and excellent metabolic ability, they show potential application prospects in the field of tumor imaging and treatment. However, BBTD-like derivatives are inherently hydrophobic and have no tumor-targeting ability. Polyethylene glycol (PEG) is often used for functional modification to prepare fluorescent polymer nanoprobes. Targeting groups are then attached to the surface of nanomaterials, so as to achieve partial targeted imaging of tumors and rapid metabolic clearance from organs such as the kidney or liver [148,149,150]. Dai et al. first used BBTD as the acceptor and triphenylamine (TPA) as the donor to prepare the compound CH1055 with a typical D–A–D structure. CH1055-PEG was prepared after modification with polyethylene glycol (PEG), and its maximum emission wavelength in water was 1055 nm. Experiments have shown that the imaging effect on mouse blood vessels and lymph is better than that of the commercial dye indocyanine green ICG, and about 90% of the material can be metabolically cleared from the kidney within 24 h [151]. In recent years, based on the compound CH1055, it has become possible to prepare a series of CH1055 derivatives by using different group modifications, such as the water-soluble CH-4T [152] prepared using sulfonation modification. With the help of follicle-stimulating hormone (FSH) modification, it is prepared into FSH-CH [153] and so on.

## 6. Summary and Outlook

Early diagnosis and treatment of cancer can greatly reduce its incidence and mortality. As a noninvasive and visualized diagnosis and treatment method, bioluminescence imaging technology, combined with traditional imaging technology, provides reliable imaging means for the early diagnosis of cancer. This article systematically introduces the research and development status and application prospects of fluorescent polymer nanomaterials based on rare earth luminescent materials, semiconducting polymers, and small organic molecules from recent years. Various types of luminescent probes have been developed for tumor diagnosis. However, due to the hydrophobic nature of the luminescent probe itself, when it is further functionalized, most of the luminescent materials are encapsulated inside the polymer nanoparticles using physical packaging. The method is simple to operate and has good universality. However, there are also defects, for example, the luminescent material leaks easily from the nanoparticles, the particle size of the nanomaterials prepared in different batches is not uniform, and the encapsulation rate of the materials is different. Therefore, searching for efficient functionalization methods for luminescent materials is still one of the core issues in the design and development of new luminescent materials.

In the decades since the development of polymeric nanomaterials in the field of optics, researchers have conducted a lot of work on synthesis control and material selection. While making breakthroughs, there is still demand for both high-performance and multifunctional materials. This also poses a higher challenge to the application of fluorescent polymer nanomaterials and biological fields. This is mainly reflected in the following aspects:

(a) Fluorescent materials should be combined with current scientific theories and technologies. With advancements in science and technology, optical materials, as a traditional research field, can be further developed in the direction of diversification, high technology, and high performance when combined with advanced theory and technology. Thus, new subject areas are created [154,155].
(b) Quantification of the structure–property relationship of multifunctional fluorescent polymer nanomaterials: Just as researchers have studied the “wetting” and “dewetting” of polymer-grafted core–shell particles in the matrix [156,157], essentially, the quantification of the effect of this structure on light transparency has developed from simple conclusions to a formalized theory. This method can be continued and expanded upon for other properties, forming quantitative structure–effect relationships for various properties.
(c) The trade-off between key properties in the design of optical materials: The discussion in this article maintains a relatively consistent train of thought with most of the work. That is, starting from the material with a certain performance, the study on the impact of the structure on the improvement of this single performance, while ignoring the impact on other performances, or even the actual application environment. This is not conducive to the multifunctionalization of optical materials. Starting from key structural factors such as particle size, grafting, and loading in the structure of hybrid materials, the synergistic effects of a certain change in structure on various properties can be discussed to form an application-oriented material performance trade-off strategy.
(d) Attention paid to the development of new optical functional materials: In addition to paying attention to the development of new photofunctional inorganic materials or polymer materials, we also need to pay attention to the development of new hybrid material systems. Carbonized polymer dots (CPDs) are a new type of nanofunctional elementary material that represent a new system of polymer nanohybridization [158,159]. In recent years, the advantages of this material have been reflected in its luminescent properties, and its synthesis process is considered to be a crosslinking carbonization process involving small molecules or polymer precursors. After several years of exploration, a family of CPDs with various luminescent properties such as full-color luminescence and narrow half-peak width emission has been obtained. Recently, Yang et al. further designed and applied this material to a material with both light transmission and mechanical properties, realizing the application of CPDs in the field of transparent optical films [160]. In addition, CPDs have also demonstrated their contributions in multiple fields such as imaging, sensing, and energy [161]. Their advantages such as low toxicity, environmental friendliness, and structural designability [162] lead us to believe that the introduction of new material systems such as CPDs will provide more excellent performance and broader application prospects for multifunctional fluorescent polymer nanomaterials.
(e) In order to promote the further application of fluorescent polymer nanomaterials in biomedicine, future research work can be optimized and expanded in the following aspects. At present, most light-emitting polymers have low light-emitting performance in NIR-II. The development of NIR-II light-emitting polymer materials with higher luminous intensity or photothermal efficiency using DA structure adjustment combined with theoretical calculations is still one of the important research directions for the future. Efficient enrichment of luminescent nanofunctional materials in tumor sites is another key to improving the efficiency of tumor diagnosis and treatment. Using rational molecular design, targeting groups can be effectively bonded to polymer chains to prepare light-emitting polymers with targeting functions, which is of great significance for the precise diagnosis and treatment of tumor sites. A single treatment for tumors is gradually being replaced by multimodal treatment. The therapeutic effect on tumors can be improved by constructing a multifunctional nanodiagnosis and treatment platform with properties such as chemotherapy, photodynamic therapy, or photothermal therapy.
(f) Internal/external stimuli-responsive fluorescent polymer nanoparticles that can be used in theranostics and sensing applications cannot be ignored either [163]. In particular, they respond to internal stimuli, including redox, pH, and enzymes, and external stimuli, including temperature, light, and magnetic fields, for drug delivery and sensing applications [164,165]. In terms of generating stimulus-responsive signals, these signals allow for amplification and easy detection of biologically relevant events. More detailed modeling of the photophysical properties of existing materials and their properties will provide decisive input for designing better performing NPs.

## Figures and Tables

**Table 1:** Comparison of different preparation strategies for fluorescent polymer materials.

Synthesis Technology		Advantages	Disadvantages
Living/controllable synthesis of amphiphilic block polymers	ATRP	The reaction temperature is mild, the operation is simple, and it is easy to industrialize.	The intermediate process is completely uncontrolled; the amount of transition metal complex is large; the aging of the polymer.
	RAFT	It has a wide range of applications, good polymerization ability, and the molecular weight of the obtained polymer is uniform.	Few applicable monomers, limited scope of molecular design, expensive.
Physical package	Nanoprecipitation	Simple operation, fast, high reproducibility, good dispersion of colloidal nanoparticles and easy functionalization.	There are fewer types of polymers, and the process of particle growth is not easy to control.
	Microemulsion method	Narrow particle size distribution, controllable, good stability.	Surfactants are difficult to remove and have large particle sizes.
	Self-assembly method	It is very convenient to prepare various exotic three-dimensional structures; it is also possible to prepare porous materials that inherit the original morphology and structure	Unstable under physiological conditions.
Covalent linkage	PISA	The process is simple, the price adjustment is gentle, and nano-medicine can be prepared in one step.	The operation is complicated, the reaction takes a long time, and the concentration of the prepared nanoparticles is low (≤1 mg/mL), which makes it impossible to achieve large-scale mass production.
	Precipitation polymerization	The particle size of the polymer is uniform and clean, the viscosity of the polymerization system is low, and no surfactant and stabilizer are needed.	Low microsphere yield and high solvent toxicity.

**Table 2:** Biotoxicity and biological system applications of different fluorescent nanomaterials.

Fluorescent Nanomaterial Type	Intrinsic Material Toxicity	Materials	Biological System
Carbon dots	Low	C-dots, PEG stabilized	Mice
Carbon nanotubes	Low–medium	Many types of CNTs	Various in vitro/in vivo
Dendrimers	High	Various dendrimer types	Various in vitro/in vivo
Doped graphene QDs	Medium	N-doped graphene quantum dots	Red blood cells
Fluorescent beads	Medium (polymer)	Polystyrene nanoparticles	Endothelial cells
	Low (silica)	Silica nanoparticles	Epithelial cells and fibroblasts
Fluorescent proteins	Medium	Red fluorescent protein	HeLa cells
Graphene oxide	Medium	Graphene oxide	Various in vitro/in vivo
		Graphene oxide	Red blood cells
Organic dyes	Medium	Various organic fluorophores	Various in vitro/in vivo
Metal clusters	Medium	MPA or GSH stabilized Au clusters	Colonic epithelial cells
		GSH and BSA stabilized Au_2_5 clusters	Mice
Nanodiamonds	Low	Detonation nanodiamond	Various in vitro/in vivo
		Various diamond types	Human liver cancer and HeLa cells in vitro
		Detonation nanodiamond	Human embryonic kidney cells and Xenopus laevis embryos
P-dots	Medium	Quinoxaline based polymer, STV conjugated	Zebrafish embryo
		Polybutylcyanoacrylate	HeLa and human embryonic kidney cells/rats
Quantum dots	High	CdSe–ZnS; PEG, BSA or polymer stabilized	Rats
		CdTe	Mice
		Several types	Various in vitro/in vivo
		Several types	Various in vitro/in vivo
Rare earth nanoparticles	Medium-high	UCNPs, NaYF_4_:Yb,Tm, polyacrylic acid coated	Mice
		UCNPs, NaYF_4_:Yb,Tm	HeLa cells, caenorhabditis elegans
		DCNPs, Gd_2_O_2_S:Tb^3+^	Human peripheral blood mononuclear cells, human-derived macrophages, HeLa cells

**Table 3:** Common fluorescence excitation and emission wavelengths.

Fluorescent Substance	Excitation Wavelength		Emission Wavelength
	EX nm	EX (sub)	EM nm
Alexa Fluor 532	532		554
Cy3	550		570
DsRed	557		579
EtBr	300	518	605
FITC	490		525
Gel Green	250	500	530
GFP	488		507
mCherry	580		610
SYBR Gold	495		540
SYBR Green I	498		522
SYPRO Red	550	300	630
SYPRO Ruby	280	450	620
TagRFP	555		583
Gel Red	270	510	600

**Table 4:** Several photophysical properties of PFBT Pdots, IgG-Alexa 488, and Qdot 565 [138].

Probes Size	PFBT ~10 nm	IgG-Alexa 488 ~1 nm	Qdot 565 ~15 nm
Abs/FL λ_max_ (nm)	460/540	496/519	UV/565
ɛ (M^−1^cm^−1^) λ = 488 nm	1.0 × 10^7^	5.3 × 10^4^	2.9 × 10^5^
Quantum yield (%)	30	90	30~50
Fluorescence lifetime (ns)	0.6	4.2	~20

## Data Availability

Not applicable.
